# Supplementary material for: Rewired RNAi-mediated genome surveillance in house dust mites
Source: PLoS Genet. 2018 Jan 29;14(1):e1007183. doi: 10.1371/journal.pgen.1007183 (PMC5805368; doi:10.1371/journal.pgen.1007183)
Supplement: S1 Text — (DOCX) [file pgen.1007183.s001.docx]

**Supplementary Text**

**Bulk Collection of Mixed Stages Mites**

Isolation of American house dust mites *Dermatophagoides farinae* (mixed stage, male and female) for extracting genomic DNA and complete RNA was accomplished using a salt bath procedure. Adding mites to a flask of high molarity NaCl (3M) solution caused animals to float due to the mites being hypo-osmolar to the liquid. After adding the animals along with food particles (fish food), the solution was swirled on magnetic stir plate for five minutes to separate animals from food, which sinks to the bottom of the flask. Animals normally tended to gather near the edges of the flask on top of the solution. Then they were separated from the flask using filter paper, dried, and collected in conical tubes with paint brushes.

**High Molecular Weight (HMW) Genomic DNA Extraction**

Mixed stages homogenized mites were gently re-suspended in 5 ml lysis buffer (7M Urea, 2% SDS, 50mM Tris pH 7.5, 10mM EDTA and 0.35M NaCl) and 5ml of 1:1 Phenol/Chloroform. The mixture was rotated slowly on a nutator for 30 min and after that centrifuged for 10 min at 18000 rpm at 20^o^C. The aqueous phase was separated and re-extracted twice following the above-mentioned process and then two volumes of ethanol was added and the mixture was centrifuged at 18000 rpm for 10 min at 20^o^C, which resulted precipitation of the DNA. The pellet was re-suspended in 3 ml of TE (10mM Tris 1mM EDTA pH 8.0). To avoid shearing of DNA, all steps were handled very gently. To the re-suspended DNA, 3g CsCl and 0.3ml of 10mg/ml Ethidium Bromide (EtBr) were added and the mixture was centrifuged at 45K rpm for 16 hrs at 15^o^C. The DNA band was collected and EtBr was removed by extraction with CsCl saturated butanol. Then DNA was diluted three-fold with TE followed by addition of 1/10 volume 5M NaCl and finally the DNA was precipitated with two volumes of ethanol. After centrifugation the pellet was washed in 70% ethanol and re-suspended in TE. This HMW DNA was used for long read PacBio sequencing.

***In vitro* Transcription of dsRNA**

~500 nt exonic region was amplified by PCR for all three Dicers (Dcr1, Dcr2, Dcr3) and Derf1 allergen using *Taq* DNA polymerase, which adds an extra adenine (A) nucleotide at the 3’ end of reaction products. Amplified DNA was used in a TA cloning strategy and ligated to pGEM®-T Easy vector (Promega). After sequences were confirmed, the inserted regions were amplified by PCR using primer pairs that added T7 promoter sites to fragment ends. *In vitro* transcription was carried out by MEGAscript T7 Transcription Kit (Thermo Scientific), followed by precipitation of synthesized RNAs by LiCl. Precipitated RNAs were resuspended in water and incubated at 95^o^C for 2 minutes in heat block to denature the complementary RNAs. After denaturation, the heat block was switched off to ensure slow decrease of the temperature so that the complementary RNAs could anneal each other. After 1 hour dsRNAs were stored in -80^o^C.

**Northern Blot**

20 µg total RNAs were ran for each sample in 12.5% sequence gel. Gel was transferred onto Nylon membrane in 0.5X TBE in 10V 300mA for 1 hour in cold room followed by UV-crosslink and drying at 80^o^C for 10 min. After that the membrane was prehybridized in hybridization buffer (5X SSC, 1mM EDTA, 2X denhardt’s, 1% SDS, 2% dextran sulfate, 30 µg/ml ssDNA) for 30 min at 40^o^C. RNA probes were radiolabeled by adding Gamma-ATP (6000Ci/mmol) using T4 Polynucleotide Kinase (T4 PNK). Probe hybridization was carried out in 40^o^C overnight. Membrane was washed in 2X SSC, 0.1% SDS for 2 hours and exposed to X-ray film.

**Western Blot**

Animal lysate was prepared in standard RIPA buffer (50 mM sodium chloride, 1.0% NP-40, 0.5% sodium deoxycholate, 0.1% sodium dodecyl sulfate, 50 mM Tris, pH 8.0) and 20µg lysate was run in 8-20% precast gradient polyacrylamide gel (Thermo Scientific). Then western blot was performed using mouse anti derf1 primary antibody (Df10, Novus Biologicals USA).

**Analysis of Small RNA Datasets**

High throughput sequencing data in fastq format was acquired with an illumina Nextseq 500 and downloaded from the illumina basespace service. Reads were clipped using fastx_clipper to remove adapters. Bowtie was used to map small RNA reads to genome indexes or indexes of specific regions [1, 2]. Regions of high expression were identified by mapping with bowtie2 followed by conversion to a bedgraph file, which was filtered for coverage > 1000 [3]. Nearby regions were merged together to yield coordinates of high expressed loci.

To assess expression levels, biogenesis, or strand bias bowtie was used to capture either multi-mapping or uniquely mapping reads. In order to isolate reads specific to different regions (mRNA, TE, ncRNA–rRNA/tRNA/U6), reads were first mapped to an index of sequences of non-target sequences. For example, reads that were used to map to TE loci were a library subset that failed to map to mRNA and ncRNA sequences. Mapping to unknown loci used reads that failed to map to mRNA, TE, and ncRNA. Output .sam and .fastq files were generated for each category [1]. An awk command was used to sort the mapped reads based on size followed by uniq command to determine number of reads in each size category. A python script was executed on .sam files to calculate z-scores for read overlap probabilities [4]. The sequences in fasta file were then used to search homologous sequence in the database using Blast2Go. RepeatMasker and HMMER were used to extract TE like sequences from the *D. farinae* genome, which were analyzed in the same pipeline (S1 Fig) [5]. Coverage of TE loci was determined by calculating per base depth using samtools to isolate mapping events from high expressing loci and bedtools to determine coverage across the loci [3, 6]. Strand Bias was calculated as the ratio of the difference in coverage between strands to total coverage per strand. deepTools was used to visualize depth of reads [7].

**Gene abbreviations for S8 Fig**

**Name of Species Gene Abbreviation Accession Number**

**Cnidaria**

*Nematostella vectensis* NvecA ABZ10549

NvecB ABZ10551

*Hydra magnipapillata* HmagA Hma2.212274

HmagB Hma2.205202

HmagC Hma2.222700

**Annelida**

*Capitella teleta* Ctel ELU12939

*Helobdella robusta* Hrob 103772

**Mollusca**

*Lottia gigantea* Lgig 61365

**Platyhelminthes**

*Schmidtea mediterranea* Smed1 ASA.00018.01

Smed2 mk4.000125.07.01

*Schistosoma mansoni* Sman1 Smp_169750.1

Sman2 Smp_033600

*Schistosoma japonicum* Sjap1 Sjp_0069770

Sjap2 Sjp_0043700

*Echinococcus granulosus* Egra1 EgrG_000085200

Egra2 EgrG_000181800

*Echinococcus multilocularis* Emul1 EmuJ_000085200

Emul2 EmuJ_000180900

Emul3 EmuJ_000181800

*Hymenolepis microstoma* Hmic1 HmN_000252400

Hmic2 HmN_000200100

*Taenia solium* Tsol1 TsM_000872800

Tsol2 TsM_000756400

**Nematoda**

*Caenorhabditis elegans* Cele NP_498761

*Bursaphelenchus xylophilus* Bxyl BUX_s00116.153

*Pristionchus pacificus* Ppac WBGene00096444

*Strongyloides ratti* Srat g5271

*Brugia malayi* Bmal WBGene00225287

*Trichinella s*piralis TspiA XP_003377020

TspiB XP_003375890

*Loa loa filariasis* Lloa XP_003137813

**Arthropoda**

*Daphnia pulex* DpulA EFX72380

DpulB EFX69538

DpulC EFX86072

*Pediculus humanus corporis* Phum XP_002429494

*Tribolium castaneum* Tcas1 XP_968993 1865

Tcas2 NP_001107840 1623

*Nasonia vitripennis* Nvit1 XP_001605287 1917

Nvit2 XP_001602524 1450

*Acyrthosiphon pisum* ApisA XP_001943370 1626

ApisB XP_001945890 1691

*Drosophila melanogaster* Dmel1 NP_524453 2249

Dmel2 NP_523778 1772

*Anopheles gambiae* Agam1 XP_003436256 2336

Agam2 XP_320248 1672

*Aedes aegypti* Aaeg1 XP_001652212 1658

Aaeg2 XP_001659747 2193

*Culex pipiens quinquefasciatus* Cpip1 XP_001844757 2270

Cpip2 XP_001855187 1165

*Dermatophagoides farinae* Dfar1 KY794588

Dfar2 KY794589

Dfar3 KY794590

*Sarcoptes scabiei*  Ssca1 KPM03314.1

Ssca4 KPM06069.1

*Tetranychus urticae*  Turt1 XP_015789823.1

Turt2 XP_015784164.1

**Echinodermata**

*Strongylocentrotus purpuratus* Spur XP_790894 1850

**Chordata**

*Branchiostoma floridae* Bflo XP_002610617 1868

*Ciona intestinalis* Ciona ENSCINP00000017117 1872

*Saccoglossus kowalevskii* Skow Sakowv30031161m

**Supplementary Tables**

**S1_Table: Dust mite transcriptome annotations**

(Provided in a separate file: DustMite_mRNA.bed)

**S2_Table: Annotated all TE in the dust mite genome** (provided in a separate file: DustMite_TE_ ncRNA_And_HighExpressingLoci.bed)

**S3_Table: IDEFIX TE coordinates of *D. melanogaster*** (provided in a separate file: IDEFIX_Fly.bed)

**S4_Table: Sequencing results of PacBio and Illumina** (provided in a separate file)

**Oligonucleotides**

**pGem T7 primers**

pGEM 5' T7:

TAATACGACTCACTATAGGGAGAAATTGGGCCCGACGTCGCAT

pGEM 3' T7: TAATACGACTCACTATAGGGAGAGAGCTCTCCCATATGGTCGACCTG

**Primers for dsRNA synthesis against Derf1**

DerF1CDSFwd: ATGAAATTCGTTTTGGCCATTGCCTC

DerF1CDSRev: TCACATGATTACAACATATGGATATTGTTCGATCATC

DerF1RNAiT7s: TAATACGACTCACTATAGGGTCATTGGATGAATTCAAAAACCG

DerF1RNAiT7as: TAATACGACTCACTATAGGGTTGGTATTGTATCGCCGTGAC

**Primers for dsRNA synthesis against Dicers**

Dcr1 F: GACGAACAACTTTATCGAGATGCAG

Dcr1 R: AACAGACCATCCAAAATCTAACTTGGG

Dcr2 F: TTAACCGACCATCGATTAGTATCGG

Dcr2 R: GTGTTTTATTGTCCATATCATGAAAATCAGC

Dcr3 F: GTTGTTACACCCGATATTTTGTTGG

Dcr3 R: CTTATGAATTTTCATAAATACAAGCTG

**Primers for qPCR**

18S_rRNA F: GGCTACCACATCCAAGGAAGG

18S_rRNA R: GCATAAGCGAAGCCCGTATTG

Derf1qPCR F: ATGCCGACGACCAAATTCGC

Derf1qPCR R: CGGCAATAGCTGTGTGTGTTTGAG

Dcr1 qPCR F: TTACCGACGAAAAACGTCAGC

Dcr1 qPCR R:   GACGATCGAAACGAAGTGAAG

Dcr2 qPCR F: GATTACTGGTGATCATAATCCGG

Dcr2 qPCR R: CATATAATATTGCTGGTGTCAG

Dcr3 qPCR F: GTTTCGGAAAGAACGGATGC

Dcr3 qPCR R: CCGCAAATAAAACCTGGTTTAAG

HE_TE2 F: GTATTATGTCCAACACTTTCCAGTGG

HE_TE2 R: GATTTGGTCGATTGTATCATGGCAC

HE_TE6 F: GCGGAGGAAAAGAAACCAAATGGG

HE_TE6 R: GCGTTCAAGAGATGCGGCGTG

HE_TE10 F: GGTGGTTTATTCAAGCTCCTGATG

HE_TE10 R: CTCAATGCCGTTGTATTGAATTTTCGG

HE_TE11 F: AAACTTACGAAAACGCTGTCAC

HE_TE11 R: AGATCTCGATCTGTCTTCCAGG

HELITRON10 F: CTGATCTCATATTGACAGGAACGCAC

HELITRON10 R: TGGCAGTTCAGGATCTTGATCG

CHARLIE74 F: ACATGTCCTTCGCAAAACCTC

CHARLIE74 R: TGCTGCAGAGGATGAACGATAAC

Gypsy F: CATCTGATTAAATTCGTAAAGCTCTCC

Gypsy R: CAAGGGTTATTATCAGATCGAGATTGC

Unk65 F: GTTGAGTTACGCTTCGGGG

Unk65 R: CATCCGGTTTTGGTTTGTTGAC

**Primers for northern blotting**

| Dfa_ML1258siRNA2: | AGTTGCTGAGCTACTAGGTTTTA |
| --- | --- |
| Dfa_ML1258siRNA3: | GGGTTCAAGAATTATTTTCAA |
| Dfa_ML283siRNA1: | AGAATATTCAATACAGATTCT |
| Dfa_ML283siRNA2: | AGAATCTGTATTGAATATTCT |
| Dfa_ML95siRNA1: | AATGACATTACAATCCATTGGTA |
| Dfa_ML95siRNA2: | GGCTACATTGAATCCAACATTAA |
| Dfa_U6: | ACGATTTTGCGTGTCATCCTTA |
|  |  |

**Supplementary references**

1. Langmead B. Aligning short sequencing reads with Bowtie. Curr Protoc Bioinformatics. 2010;Chapter 11:Unit 11 7. doi: 10.1002/0471250953.bi1107s32. PubMed PMID: 21154709; PubMed Central PMCID: PMCPMC3010897.

2. Langmead B, Salzberg SL. Fast gapped-read alignment with Bowtie 2. Nat Methods. 2012;9(4):357-9. doi: 10.1038/nmeth.1923. PubMed PMID: 22388286; PubMed Central PMCID: PMC3322381.

3. Quinlan AR. BEDTools: The Swiss-Army Tool for Genome Feature Analysis. Current protocols in bioinformatics / editoral board, Andreas D Baxevanis [et al]. 2014;47:11 2 1-34. doi: 10.1002/0471250953.bi1112s47. PubMed PMID: 25199790; PubMed Central PMCID: PMCPMC4213956.

4. Antoniewski C. Computing siRNA and piRNA overlap signatures. 2014;(1940-6029 (Electronic)).

5. Tarailo-Graovac M, Chen N. Using RepeatMasker to identify repetitive elements in genomic sequences. Current protocols in bioinformatics / editoral board, Andreas D Baxevanis [et al]. 2009;Chapter 4:Unit 4 10. doi: 10.1002/0471250953.bi0410s25. PubMed PMID: 19274634.

6. Li H, Handsaker B Fau - Wysoker A, Wysoker A Fau - Fennell T, Fennell T Fau - Ruan J, Ruan J Fau - Homer N, Homer N Fau - Marth G, et al. The Sequence Alignment/Map format and SAMtools. 2009;(1367-4811 (Electronic)). doi: D - NLM: PMC2723002 EDAT- 2009/06/10 09:00 MHDA- 2010/01/15 06:00 CRDT- 2009/06/10 09:00 AID - btp352 [pii] AID - 10.1093/bioinformatics/btp352 [doi] PST - ppublish.

7. Ramirez F, Ryan DP, Gruning B, Bhardwaj V, Kilpert F, Richter AS, et al. deepTools2: a next generation web server for deep-sequencing data analysis. Nucleic Acids Res. 2016;44(W1):W160-5. doi: 10.1093/nar/gkw257. PubMed PMID: 27079975; PubMed Central PMCID: PMCPMC4987876.
