# Supplementary figures and images for: Rewired RNAi-mediated genome surveillance in house dust mites
Source: PLoS Genet. 2018 Jan 29;14(1):e1007183. doi: 10.1371/journal.pgen.1007183 (PMC5805368; doi:10.1371/journal.pgen.1007183)

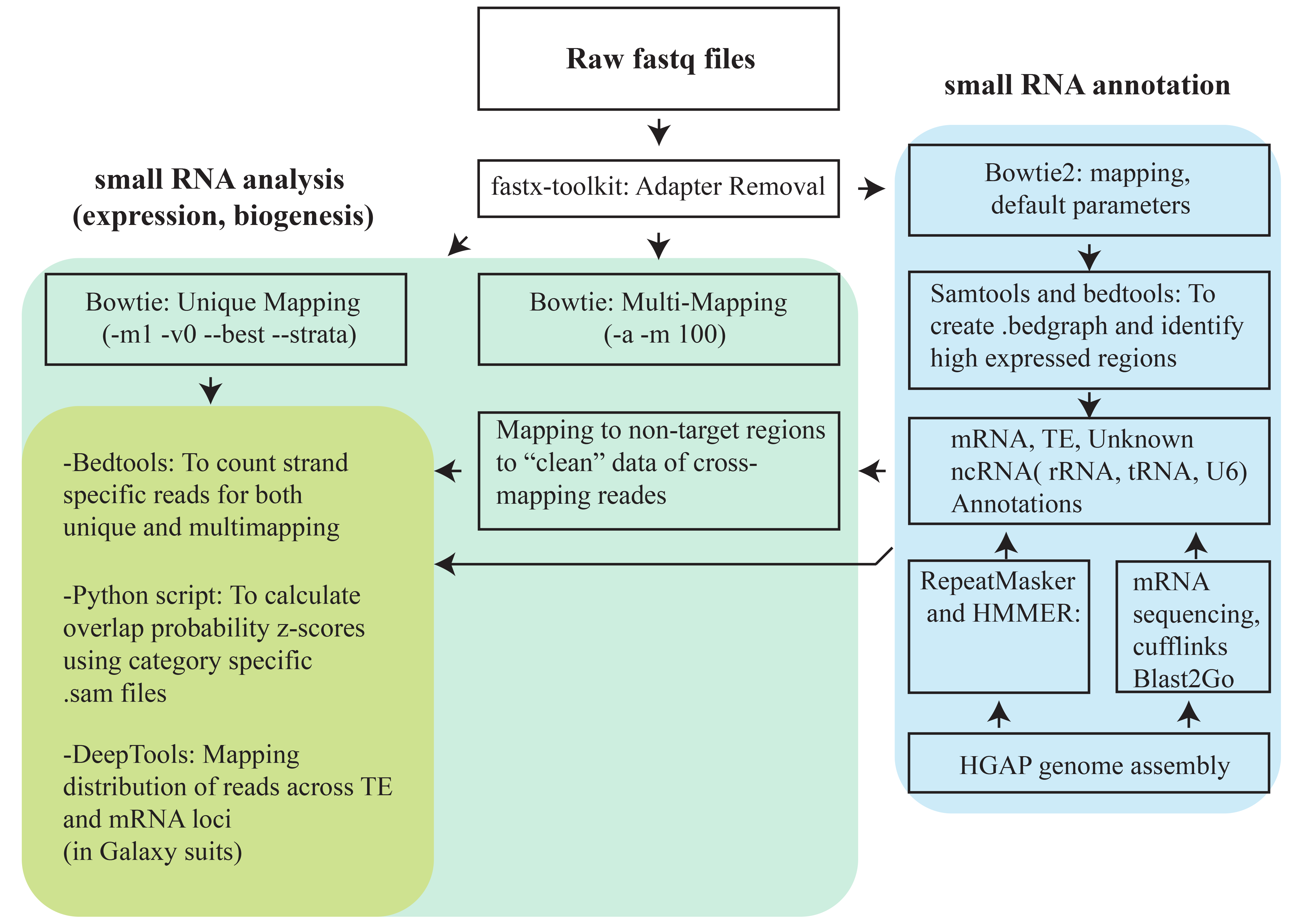

Supplement: S1 Fig — (TIF) [file pgen.1007183.s002.tif]

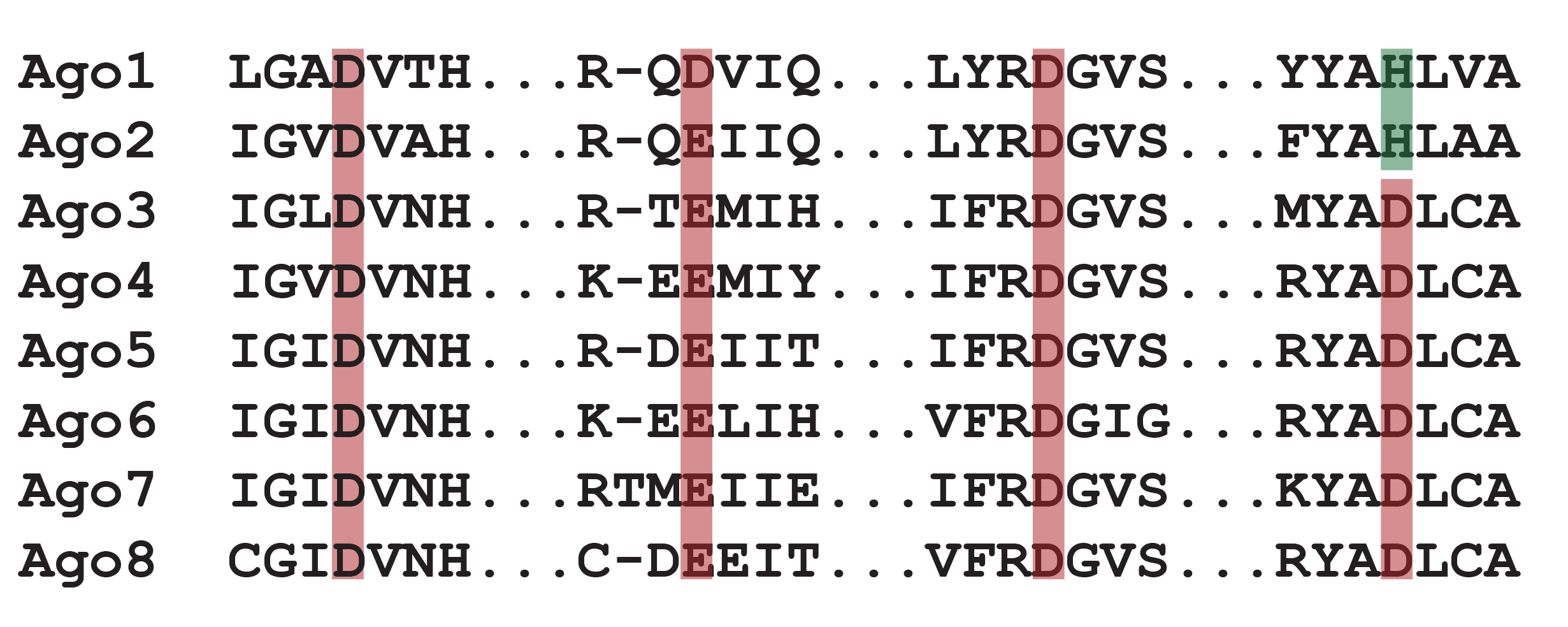

Supplement: S2 Fig — Multiple sequence alignment was carried out using clustal omega. Active site residues are highlighted in red or green. (TIF) [file pgen.1007183.s003.tif]

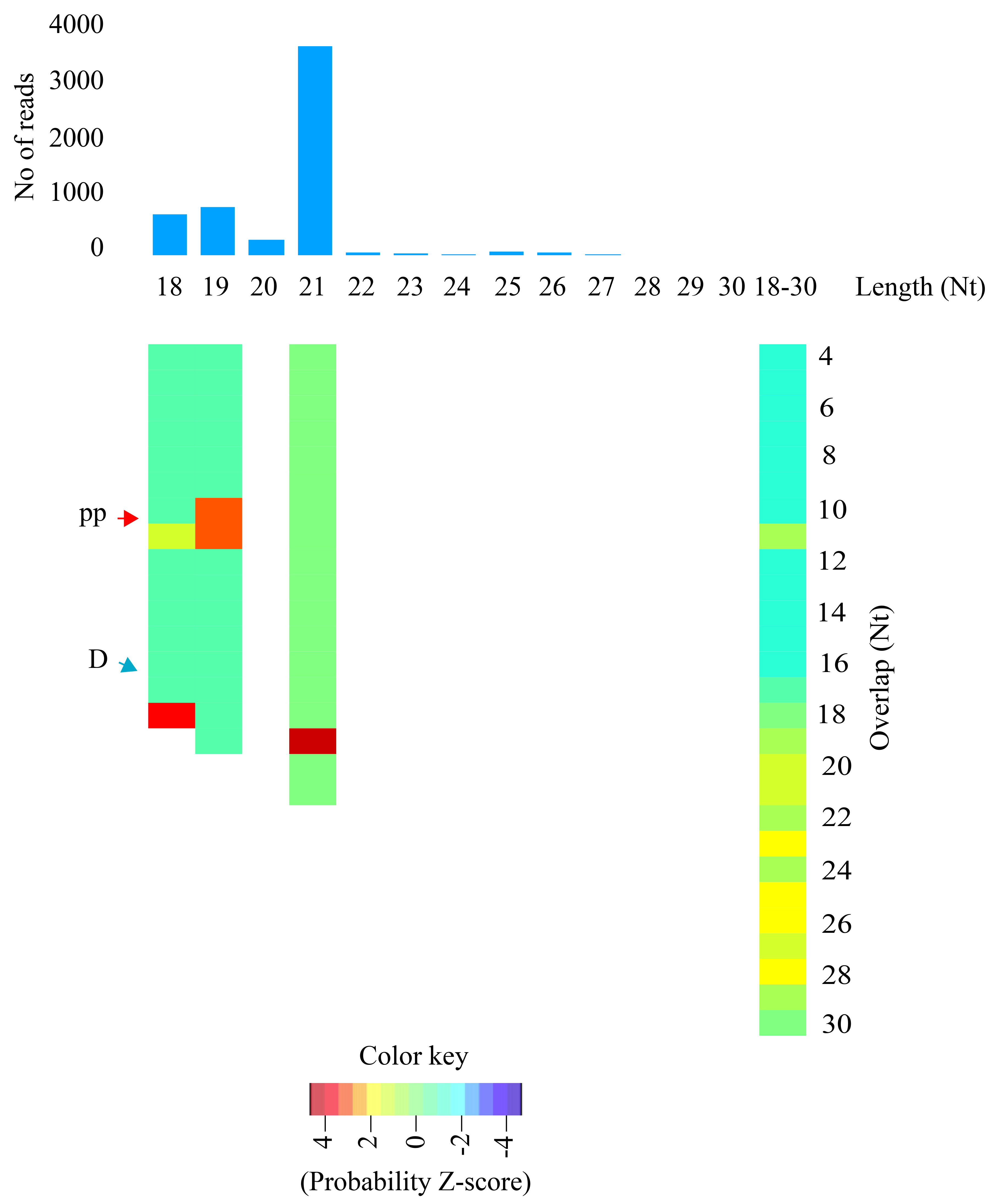

Supplement: S3 Fig — Top bar graph represents number of reads in each size. Probability z-scores were calculated for each length separately (18, 19, etc.) and together (18–30). R heatmap2 package was used to draw the heatmap. 2nt dicer processing register is shown by blue arrow “D”. Red arrow labeled “pp” shows 10nt ping-pong overlap signature. Blank areas in the heatmap are due to the absence of overlapping pairs. (TIF) [file pgen.1007183.s004.tif]

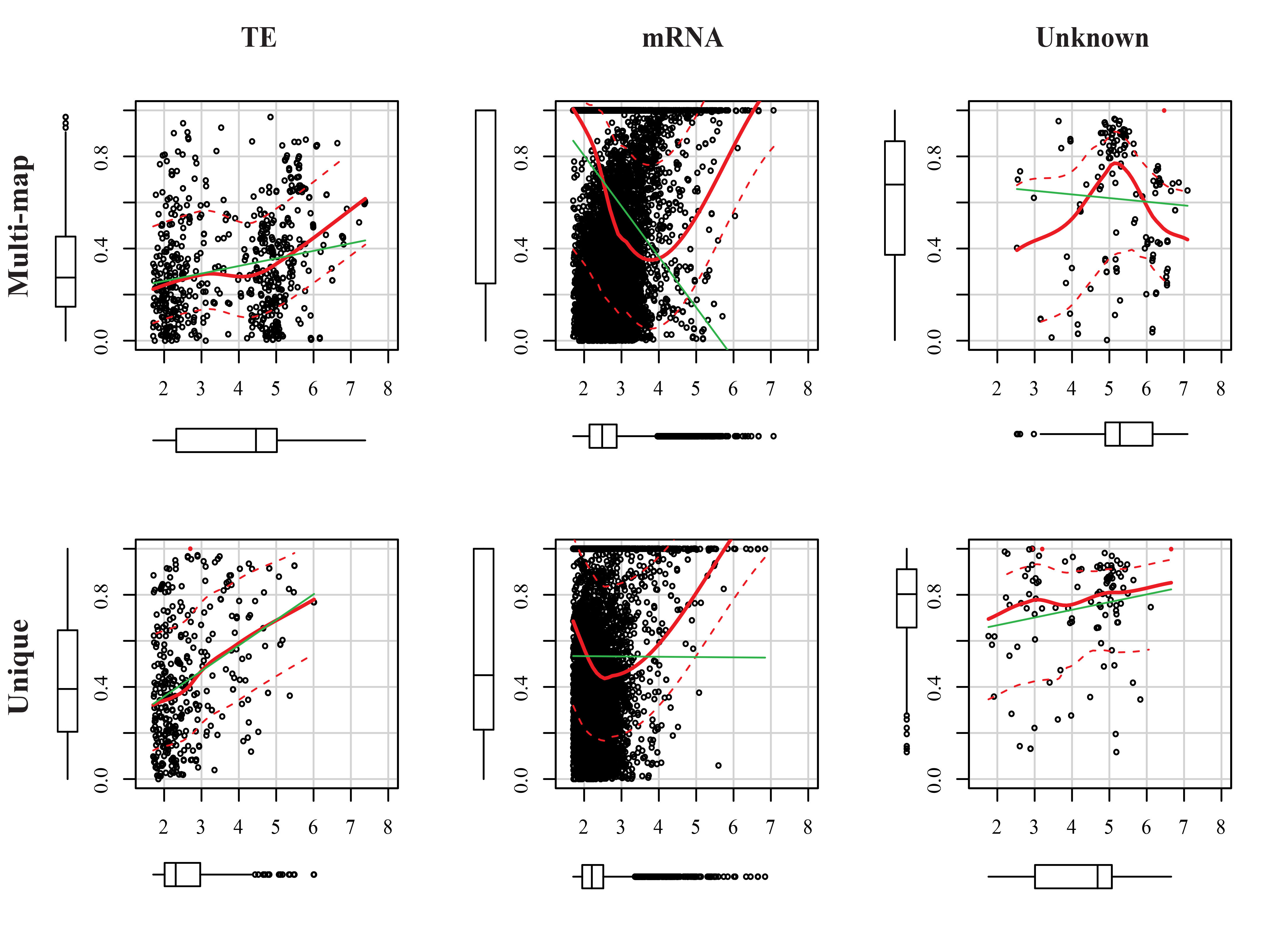

Supplement: S4 Fig — For each locus, number of mapped reads to either sense or antisense strand was determined using bedtools multicov. Strand bias was calculated by dividing the absolute difference between strand specific coverage by total converage (y-axis). Each locus is plotted by bias and log2(number of mapping reads) (x-axis). Read line indicates mean values, dotted lines standard deviation. Green regression line also plotted. Box plots on left and below show distribution of values: y-axis bias, x-axis expression. (TIF) [file pgen.1007183.s005.tif]

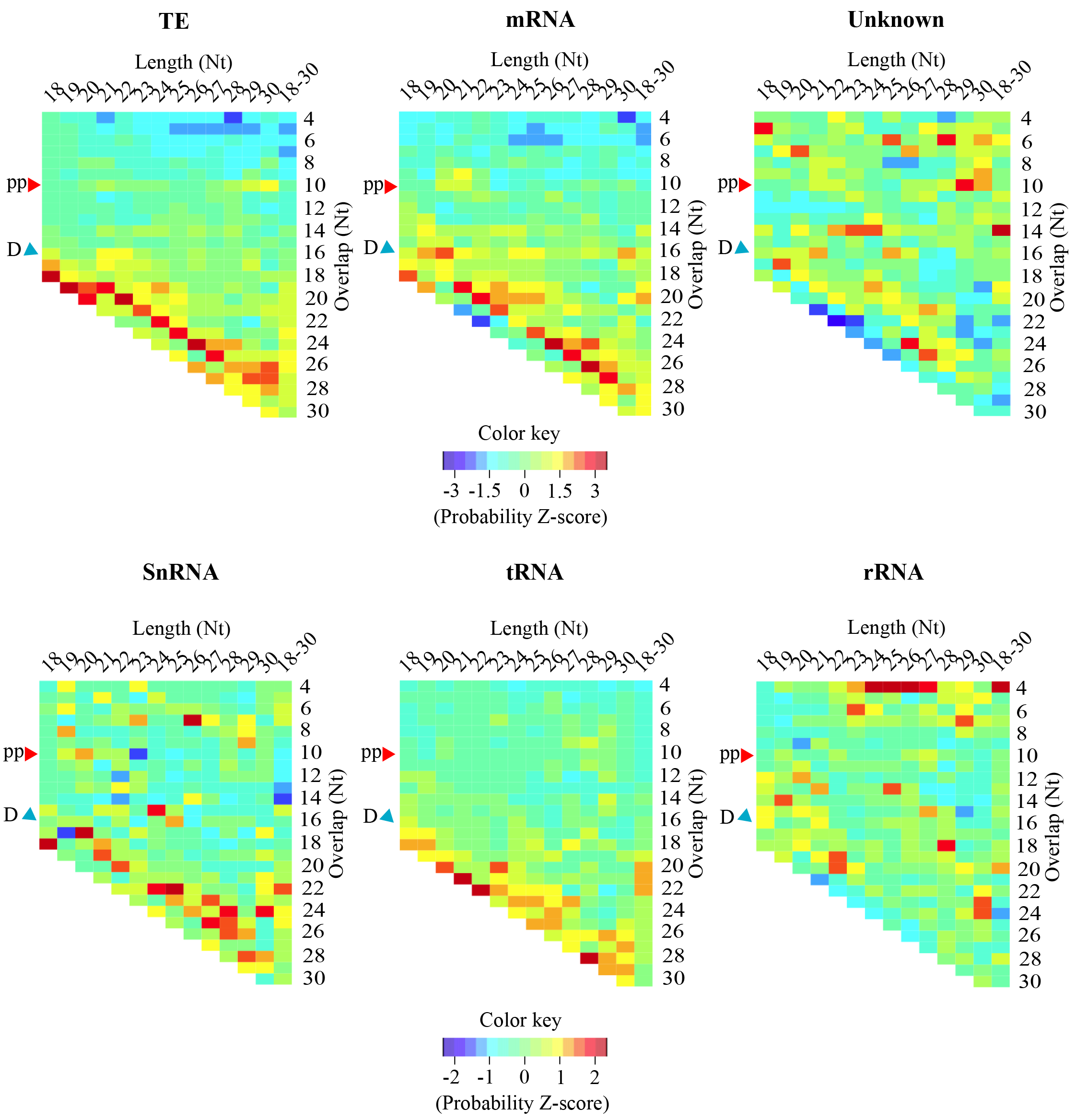

Supplement: S5 Fig — Probability z-scores, on top of maps, were calculated for each size separately (18, 19,…. 30) and together (18–30). Overlaps shown on right of maps. Heatmaps were drawn in with the R heatmap2 package. The blue arrow labeled “D” shows 2nt dicer processing register. Red arrow labeled “pp” shows 10nt overlap where ping-pong cleavage would be seen. (TIF) [file pgen.1007183.s006.tif]

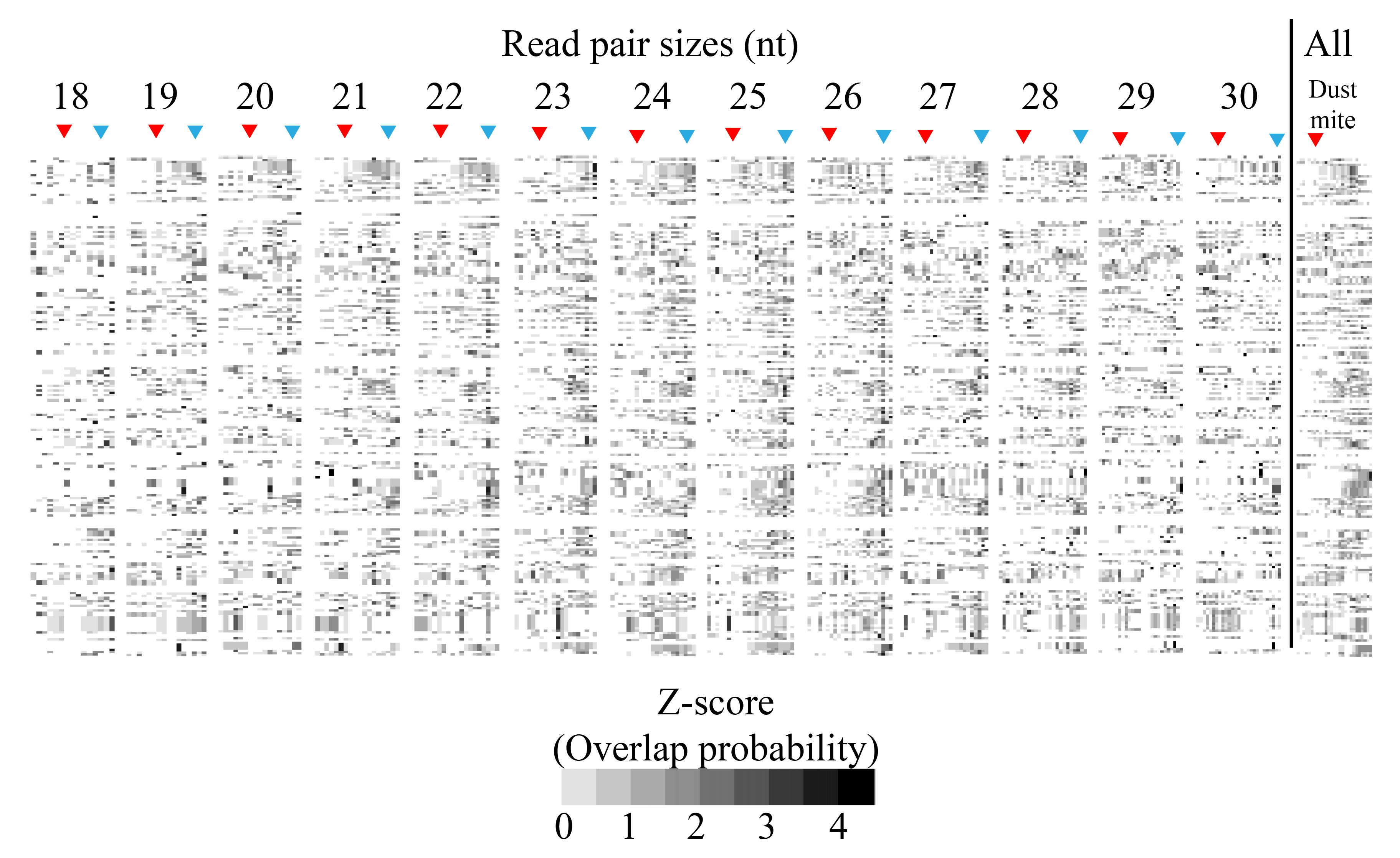

Supplement: S6 Fig — Size of read pairs indicated above the heatmaps. Blue arrows denote the expected overlap for dicer processing. Red arrows indicate expected overlap for ping pong cleavage. (TIF) [file pgen.1007183.s007.tif]

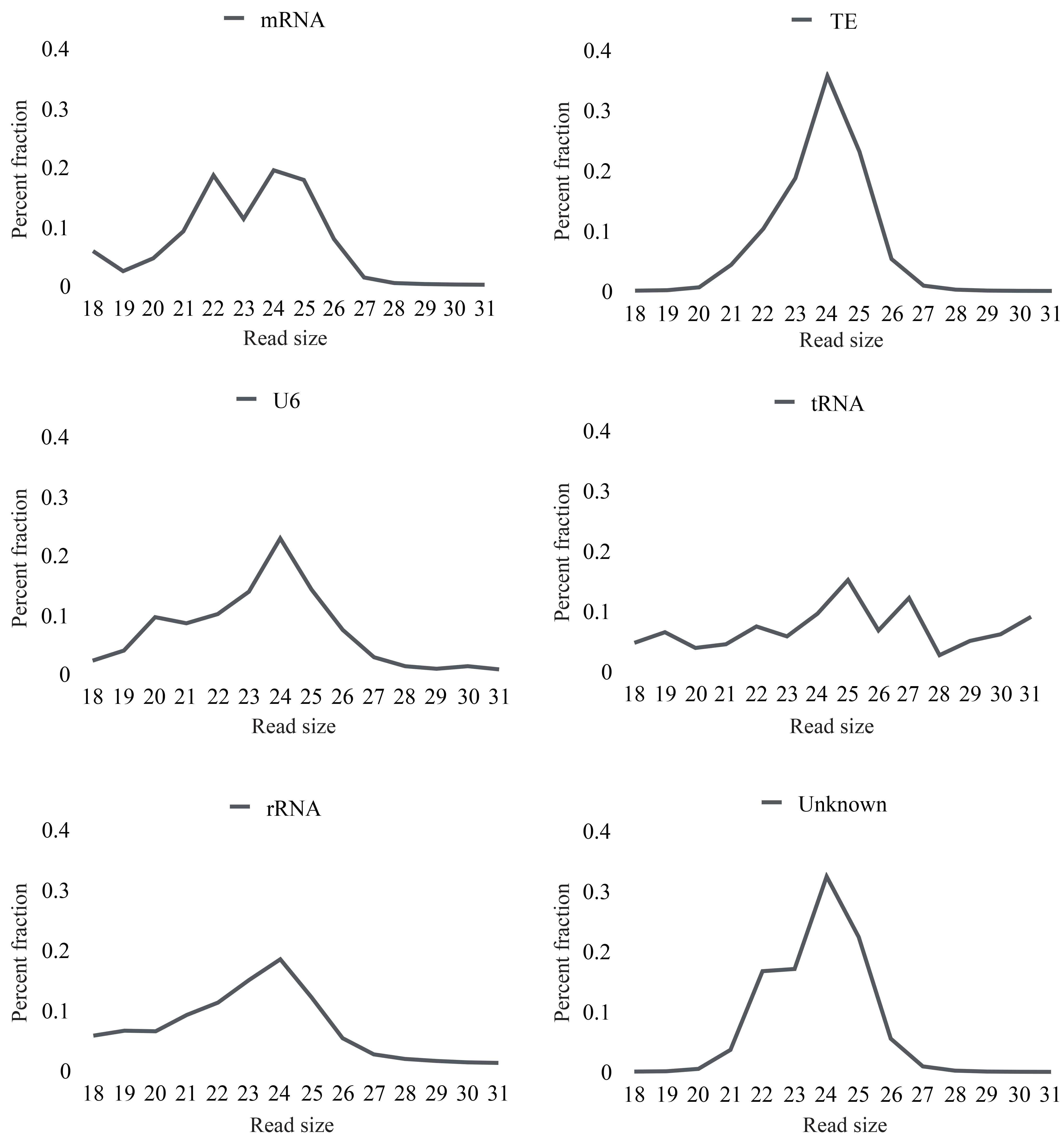

Supplement: S7 Fig — (TIF) [file pgen.1007183.s008.tif]

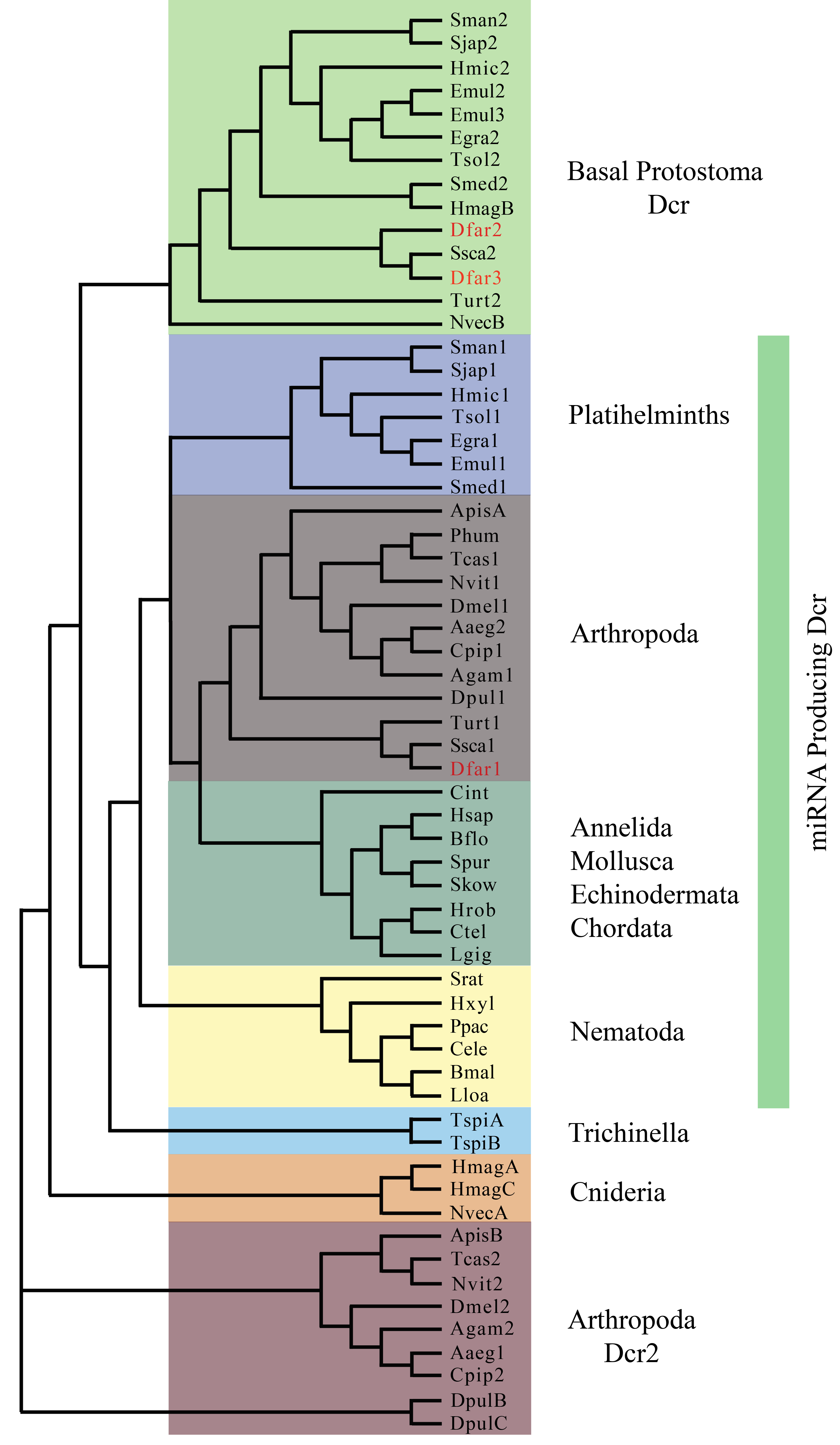

Supplement: S8 Fig — Dust mite Dicers indicated in red. Full name of the gene abbreviations can be found in S1 Text. (TIF) [file pgen.1007183.s009.tif]

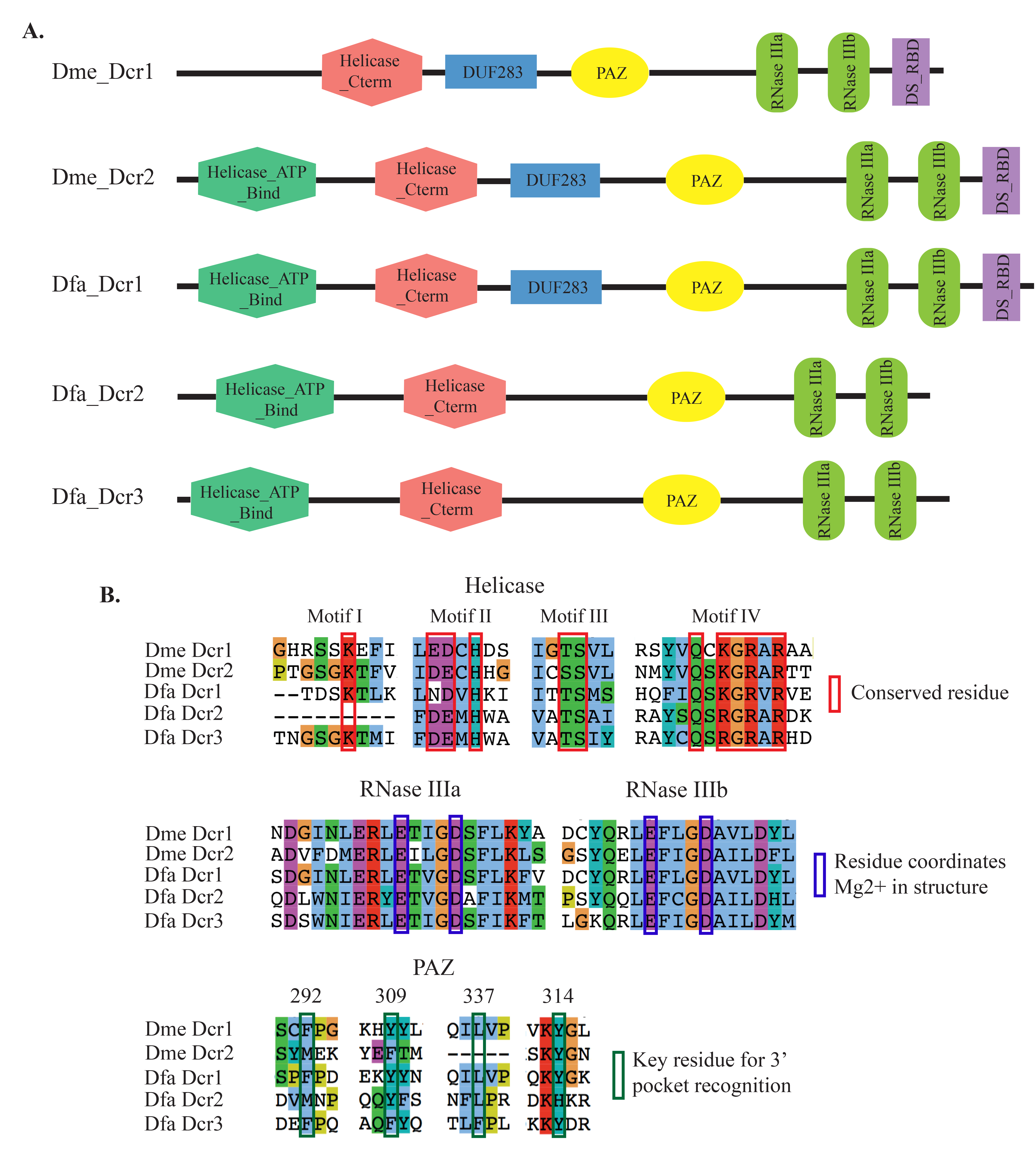

Supplement: S9 Fig — Dfa_Dcr1 (NCBI accession KY794588), Dfa_Dcr2 (NCBI accession KY794589), and Dfa_Dcr3 (NCBI accession KY794590) compared to D. melanogaster orthologs. A. Protein domain prediction of Dust mite Dicer proteins compared to Drosophila Dicers. Dicer protein domains were predicted using ScanProsite [73]. Helicase_ATP_Bind (Helicase ATP Binding domain), Helicase_Cterm (Helicase C-terminal domain), DUF 283 (dsRNA annealing domain), PAZ (Piwi-Argonaute-Zwille domain), RNase IIIa/b (RNase III domains), DS_RBD (Double stranded RNA-binding domain) B. Crucial amino acids for Dicer activity in helicase, RNase III, and PAZ domains. Multiple sequence alignment was carried out using clustal omega and alignment visualized by jalview. Amino Acid positions indicated from PAZ domain correspond to Drosophila Dicer1. (TIF) [file pgen.1007183.s010.tif]

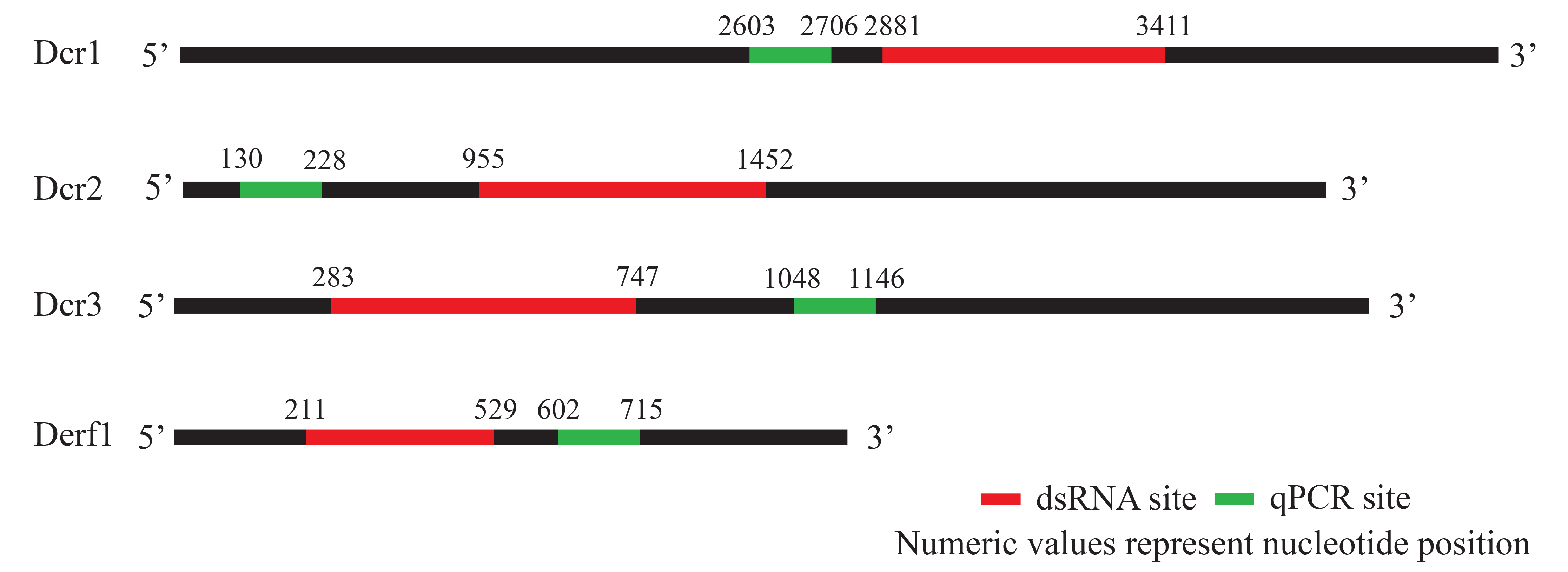

Supplement: S10 Fig — Regions used for creation of dsRNA and qPCR are shown in red and green respectively for the Derf1 and DfaDcr1-3 genes. (TIF) [file pgen.1007183.s011.tif]

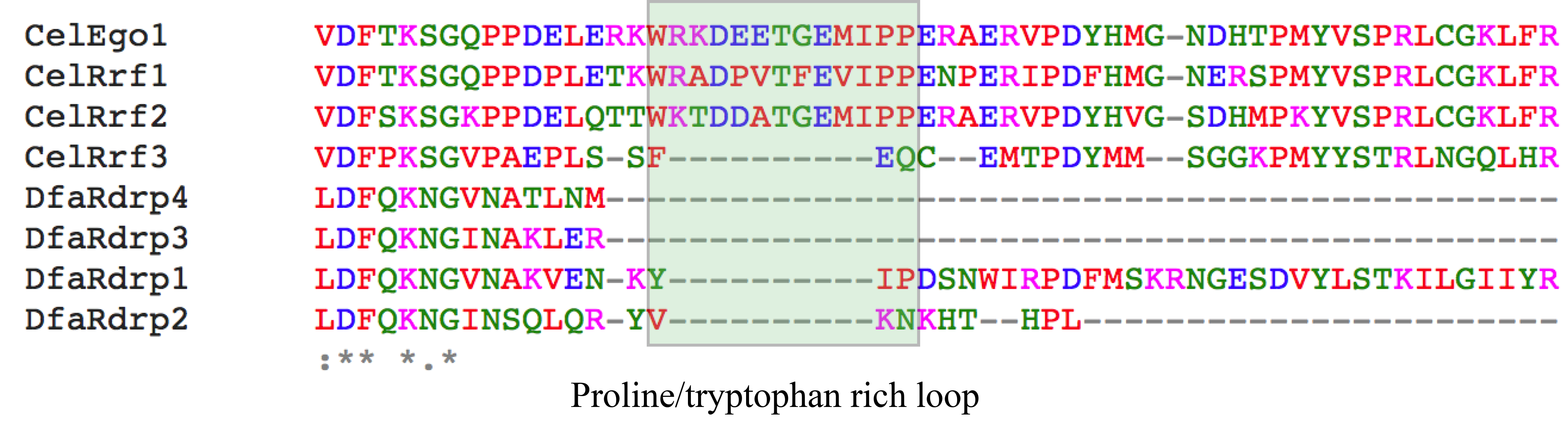

Supplement: S11 Fig — Insertion of a proline/tryotophan rich loop in RRF1/EGO1 group of Rdrp is responsible for de novo initiation of RNA synthesis, which is a property of non processive Rdrps. This group of Rdrp makes short RNAs like 22G RNA in C. elegans while processive Rdrps (RRF3 group) that do not have this loop elongate nasecent RNA for longer length. All D. farinae Rdrps do not have this loop thus are processive (RRF3 type) and synthesize longer RNAs. (TIF) [file pgen.1007183.s012.tif]

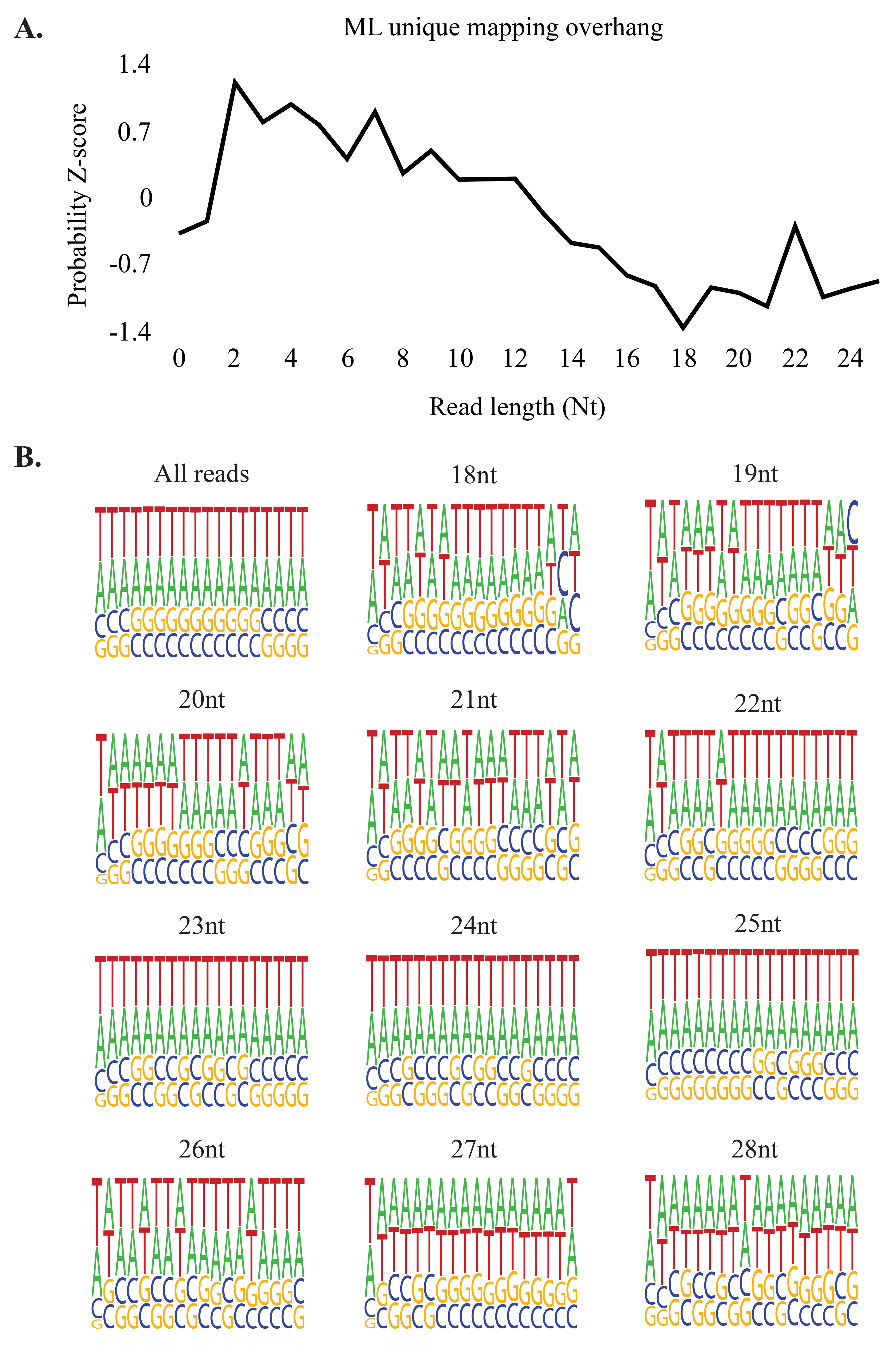

Supplement: S12 Fig — A. Overhang of reads uniquely mapping to ML-siRNA loci show a 2nt overhange, which is characteristics of Dicer processing. Overlap z-score probability was calculated using the python script for each size pair (18/18, 19/19,.....28/28) and averaged. Overlap probability was then converted to overhang probability by subtracting each overlap length from the read reangth (for example, 19 overlap probability is same as 2nt overhang probability for 21/21 pair). B. Seqlogo analysis showing nucleotide bias in ML-siRNAs. These small RNAs tend to be AT rich. (TIF) [file pgen.1007183.s013.tif]

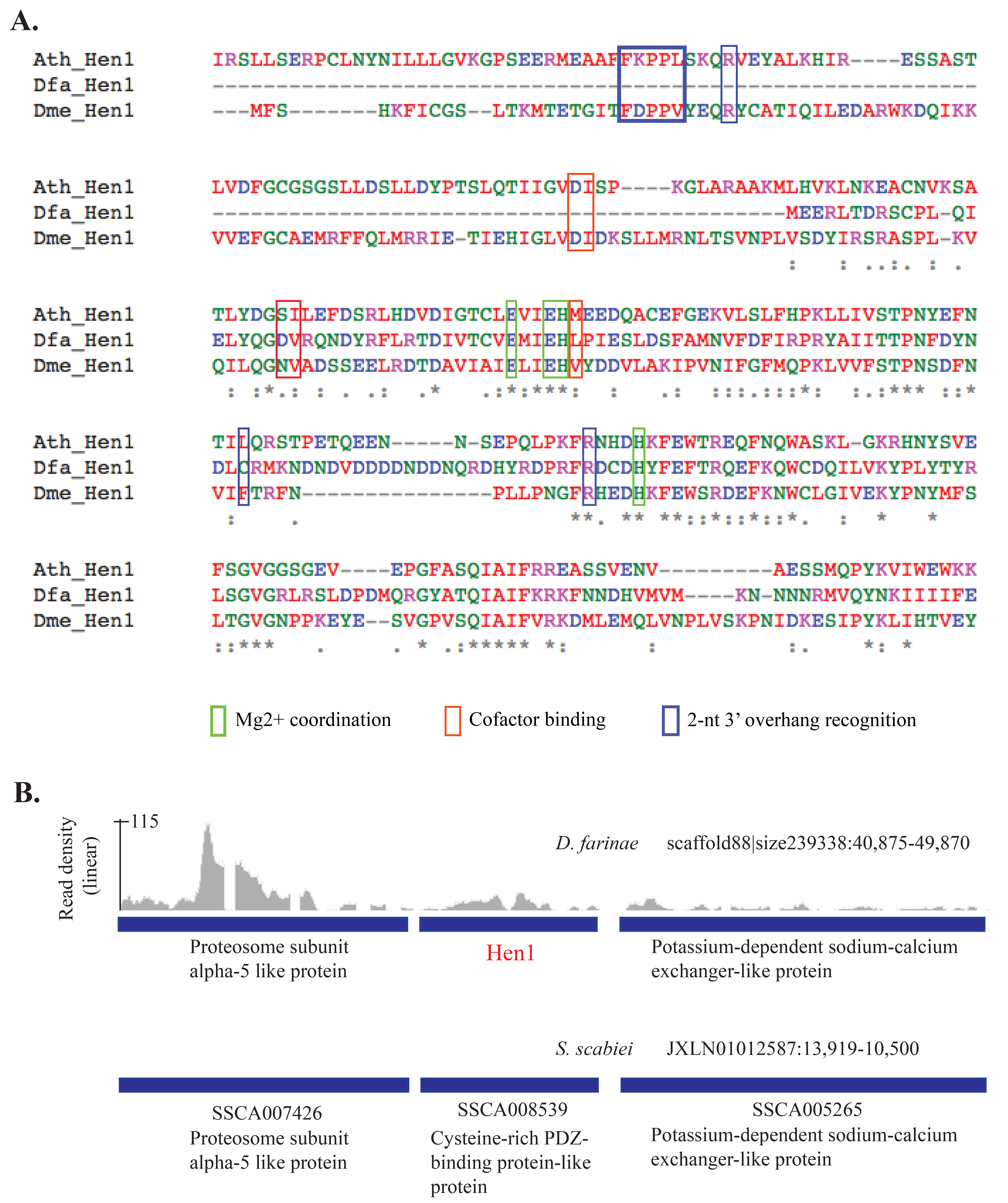

Supplement: S13 Fig — A. Sequences from Drosophila and Arabidopsis were blasted against the dust dite genome. A single Hen1 homolog was found that lacks a conserved domain involved in recognition of 2 nt 3’ overhangs found in Dicer products. B. Expression from RNA seq at the Hen1 locus and annotations of neighboring genes. Potential syntenic region from the scabies genome below showing loss of the Hen1 gene in this mite. (TIF) [file pgen.1007183.s014.tif]

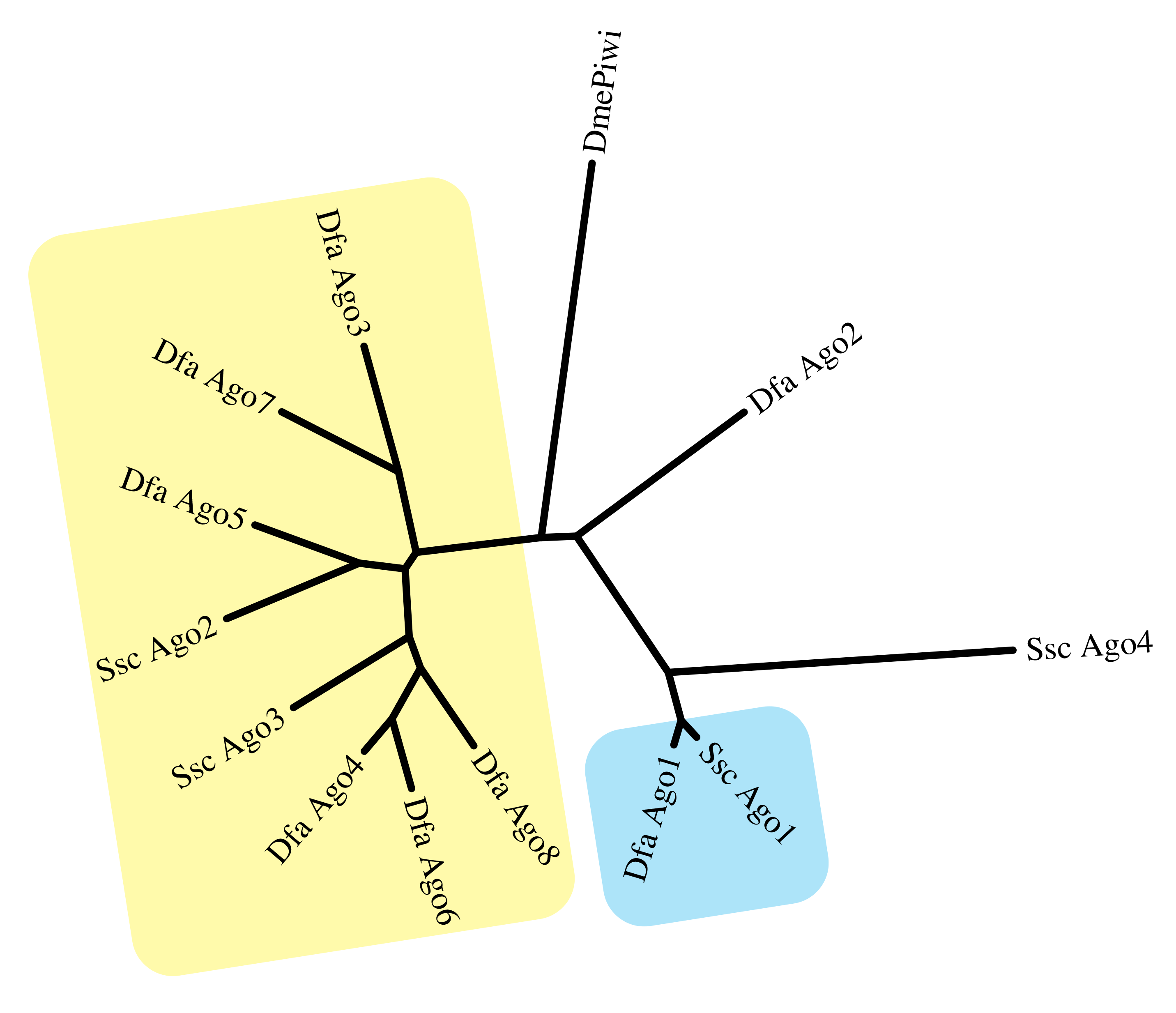

Supplement: S14 Fig — Clade containing Dust Mite specific Ago proteins described in Fig 1 highlighted in yellow. microRNA binding Agos indicated by blue. Drosophila Piwi included to demonstrate lack of clustering with this group of Ago proteins. (TIF) [file pgen.1007183.s015.tif]

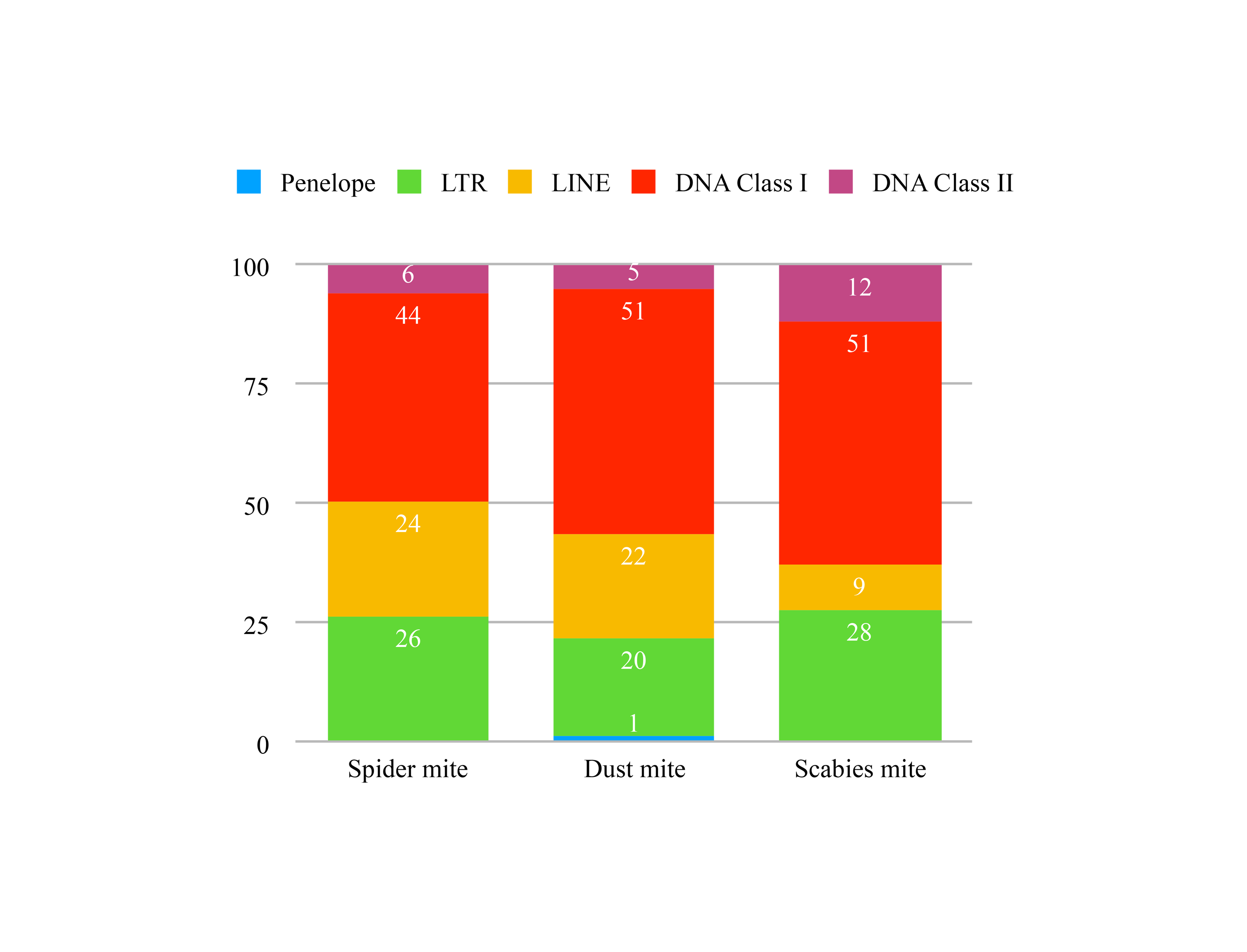

Supplement: S15 Fig — (TIF) [file pgen.1007183.s016.tif]
