# Supplementary material for: Rewired RNAi-mediated genome surveillance in house dust mites
Source: PLoS Genet. 2018 Jan 29;14(1):e1007183. doi: 10.1371/journal.pgen.1007183 (PMC5805368; doi:10.1371/journal.pgen.1007183)
Supplement: S4 Table — (DOCX) [file pgen.1007183.s020.docx]

**S4 Table: Sequencing results of PacBio and Illumina**

|  | Illumina | PacBio |
| --- | --- | --- |
| Read type | 100bp PE illumina reads | 15 flow cells of PacBio long reads |
| Number of reads | 391,617,040 | 648,931 |
| Sequencing protocol/N50) read length | 100 bp PE | 19,371 |
| Coverage | 430 | 101.26 |
